# Clinical Outcomes of Patients with Recurrent Microsatellite-Stable Endometrial Cancer in Early-Phase Immunotherapy Clinical Trials

**DOI:** 10.3390/cancers14153695

**Published:** 2022-07-29

**Authors:** Jeffrey A. How, Amir A. Jazaeri, Siqing Fu, Jordi Rodon Ahnert, Jing Gong, Bettzy Stephen, Hanna Ferreira Dalla Pria, Priya Bhosale, Amber Johnson, Ying Yuan, Funda Meric-Bernstam, Aung Naing

**Affiliations:** 1 Department of Gynecologic Oncology and Reproductive Medicine, Division of Surgery, The University of Texas MD Anderson Cancer Center, Houston, TX 77030, USA; jahow@mdanderson.org (J.A.H.); aajazaeri@mdanderson.org (A.A.J.); 2 Department of Investigational Cancer Therapeutics, The University of Texas MD Anderson Cancer Center, Houston, TX 77030, USA; siqingfu@mdanderson.org (S.F.); jrodon@mdanderson.org (J.R.A.); jinggong@mdanderson.org (J.G.); bastephen@mdanderson.org (B.S.); fmeric@mdanderson.org (F.M.-B.); 3 Department of Diagnostic Radiology, Division of Diagnostic Imaging, The University of Texas MD Anderson Cancer Center, Houston, TX 77030, USA; hrferreira@mdanderson.org (H.F.D.P.); priya.bhosale@mdanderson.org (P.B.); 4 Sheikh Khalifa Bin Zayed Al Nahyan Institute for Personalized Cancer Therapy, The University of Texas MD Anderson Cancer Center, Houston, TX 77030, USA; amjohnson2@mdanderson.org; 5 Department of Biostatistics, The University of Texas MD Anderson Cancer Center, Houston, TX 77030, USA; yyuan@mdanderson.org

**Keywords:** immunotherapy, immune checkpoint inhibitors, endometrial cancer, microsatellite stable, clinical trials

## Abstract

**Simple Summary:**

There is a crucial need to improve treatment regimens in patients with recurrent endometrial cancer. Although immunotherapy treatments have shown impressive benefit in microsatellite instability-high endometrial cancer, they have been less predictable in the majority of endometrial cancers, which are microsatellite stable. Our aim was to characterize clinical outcomes in patients with recurrent microsatellite stable endometrial cancer treated in early-phase immunotherapy clinical trials in order unravel treatment regimens that would improve response and survival. Our findings suggest that utilizing immunotherapy in combination with other non-immunotherapy agents resulted in greater duration of disease control and improved survival outcomes compared to immunotherapy only (monotherapy) or in combination with other immunotherapy agents. Future studies are needed to validate these findings.

**Abstract:**

Recurrent microsatellite stable (MSS) endometrial cancer has poor response to conventional therapy and limited efficacy with immune checkpoint monotherapy. We conducted a retrospective study of recurrent MSS endometrial cancer patients enrolled in immunotherapy-based clinical trials at MD Anderson Cancer Center between 1 January 2010 and 31 December 2019. Patients were evaluated for radiologic response using RECIST 1.1 criteria, progression-free survival (PFS), and overall survival (OS). Thirty-five patients were treated with immune checkpoint inhibitors: 8 with monotherapy, 17 with immunotherapy (IO) in combination with another IO-only, and 10 with IO in combination with non-IO therapy. Among those treated with combination IO plus non-IO therapy, one had a partial response but 50% had clinical benefit. Patients who received combination IO plus non-IO therapy had improved PFS compared to those who received monotherapy (HR 0.56, 95% CI 0.33–0.97; *p* = 0.037) or combination IO-only therapy (HR 0.36, 95% CI 0.15–0.90; *p* = 0.028) and had improved OS when compared to monotherapy after adjusting for prior lines of therapy (HR 0.50, 95% CI 0.27–0.95; *p* = 0.036). The potential beneficial clinical outcomes of combination IO plus non-IO therapy in MSS endometrial cancer should be validated in a larger study.

## 1. Introduction

Historically, there have been limited treatment options in advanced/recurrent endometrial cancer as increasing lines of systemic therapy have resulted in poorer therapeutic responses [1,2]. Recently, immunotherapy regimens have offered impressive and durable responses for patients who relapse with conventional chemotherapeutic options. In particular, pembrolizumab (anti-PD-1 inhibitor) demonstrated an objective response rate (ORR) of 57.1% among patients with microsatellite instability-high (MSI-H) tumors; a median duration of response was not reached [3]. Given this impressive response, the United States Food and Drug Administration gave pembrolizumab accelerated approval for use in MSI-H or mismatch repair-deficient (dMMR) solid tumors that have progressed on prior systemic therapy in May 2017, the first tissue-agnostic indication for a drug. Other inhibitors of PD-1 (nivolumab and dostarlimab) and PD-L1 (avelumab and durvalumab) have demonstrated similar response rates in MSI-H/dMMR endometrial tumors [4,5,6,7]

Although single-agent immune checkpoint inhibitors demonstrate efficacy among MSI-H/dMMR endometrial tumors, these agents have limited benefit in microsatellite-stable (MSS) tumors (ORR 0–12.5%) [5,7,8,9]. Additionally, the majority (approximately 70%) of endometrial cancers are MSS, and MSI-H/dMMR traits are primarily present in tumors of endometrioid histology [10,11]. These findings demonstrate an urgent need to identify better regimens and strategies to improve treatment response and overcome innate resistance mechanisms in MSS tumors. Thus, in this study, we evaluated the characteristics and clinical outcomes of patients with MSS endometrial cancer who have participated in early-phase immunotherapy clinical trials at The University of Texas MD Anderson Cancer Center.

## 2. Materials and Methods

### 2.1. Patient Population

In this retrospective cohort study design, eligible patients for study inclusion were identified through a search of the MOCLIA database from 1 January 2010 to 31 December 2019. MOCLIA is a secure institutional database that catalogues all patients, who were enrolled in Institutional Review Board (IRB)-approved clinical trials within the Department of Investigational Therapeutics at MD Anderson. Inclusion criteria for this retrospective study included the following: age ≥ 18 years, histologically confirmed recurrent endometrial cancer, non-MSI-H status, and treatment with at least one immuno-oncology (IO) therapeutic agent (defined as any drug that enhances immune-mediated anti-tumor activity). Patients were excluded if they had MSI-H tumors or did not receive at least one dose of IO therapy despite being enrolled in an immunotherapy trial. MSI status was determined either through immunohistochemical staining or polymerase chain reaction amplification. This retrospective study (PA15-0798) was approved by the Institutional Review Board at MD Anderson and the requirement to obtain informed consent was waived due to retrospective nature of this study. This retrospective study was conducted in accordance with the Declaration of Helsinki and the International Conference on Harmonization Good Clinical Practice guidelines.

### 2.2. Clinical Data Extraction

Clinical data were extracted from electronic medical records and included the following baseline patient characteristics: age, race/ethnicity, Eastern Cooperative Oncology Group (ECOG) performance status, MSI status, tumor histology, tumor grade, and number of prior lines of systemic therapy. The treatment-related information extracted included details of the immunotherapy regimen, number of immune checkpoint inhibitors, number of treatment cycles, treatment-related adverse events (TRAEs), objective radiologic response to treatment, and dates of disease progression, death, and last follow-up. Results of somatic mutational testing were also extracted and included gene panel testing via Oncomine, CMS50, FoundationOne, or Guardant360. Functional changes attributable to somatic tumor mutations were evaluated by the Precision Oncology Decision Support Team (AJ) at MD Anderson.

### 2.3. Statistical Analysis

Demographic and clinicopathologic characteristics of the study population were summarized using descriptive statistics. Clinical efficacy was evaluated through ORR, clinical benefit rate (CBR), progression-free survival (PFS), and overall survival (OS). ORR was evaluated using Response Evaluation Criteria in Solid Tumors (RECIST) version 1.1 and/or immune-related response criteria (irRC) guidelines per trial protocol [12,13]. Clinical benefit was defined as partial response (PR), complete response (CR), or stable disease (SD) as per RECIST 1.1 and/or irRC. PFS was defined from start of treatment until date of disease progression or death. Those alive and without disease progression were censored at their date of last follow-up. OS was defined from start of treatment until date of death. Those alive were censored at their date of last follow-up. The product limit estimator of Kaplan–Meier was used to estimate PFS and OS. A Cox proportional hazards model was used to compare the PFS and OS between treatment groups through calculation of hazard ratios (HRs) and 95% confidence intervals (95% CIs). TRAEs were evaluated using the National Cancer Institute Common Terminology Criteria for Adverse Events (CTCAE) v4.0 or v5.0 per respective trial protocol. The Kruskal–Wallis test and Fisher’s exact test were performed where appropriate, and a *p*-value < 0.05 was considered statistically significant. All statistical analyses were performed using Stata/MP v16.0 (College Station, TX).

## 3. Results

From the institutional database review from 1 January 2010 to 31 December 2019, we identified 35 patients with recurrent endometrial cancer, who underwent treatment in early-phase immunotherapy trials in the Department of Investigational Therapeutics at MD Anderson. Baseline clinicodemographic characteristics are shown in Table 1. The median age was 64 years. Over half of the study participants were non-Hispanic White (57.1%), with the remainder being Black (17.1%), Hispanic (14.3%), and Asian (11.4%). The predominant tumor histologic type and grade were endometrioid (42.9%) and grade 3 (85.7%), respectively. The majority (97.1%) of patients had ECOG performance status of 1, and the median number of prior lines of systemic therapy was 3 (range 1–10). There were 8 patients who received monotherapy (22.9%) and 27 who received combination therapy (77.1%). In the monotherapy group, patients were treated with single-agent anti-PD-1 (*n* = 3), anti-LAG-3 (*n* = 3), anti-CTLA-4 (*n* = 1), or anti-TIM-3 (*n* = 1) monoclonal antibodies. Among the 27 patients who received combination therapy, 17 received exclusively IO therapies (combination IO-only group) and 10 received a combination of IO plus non-IO therapies (combination IO plus non-IO group). In the combination IO-only therapy group (*n* = 17), patients were treated with combination immunotherapeutic agents as follows: anti-PD-L1/OX40 (*n* = 7), anti-PD-1/anti-CTLA-4 (*n* = 3), 4-1BB/anti-PD-L1/OX40 (*n* = 3), ICOS/anti-PD-L1 (*n* = 1), and anti-PD-1/anti-PD-L1 (*n* = 1) monoclonal antibodies. One patient was treated with an anti-CTLA-4 and a TLR-9 agonist, and 1 patient with an anti-PD-1/CSF-1 inhibitor combination. In the combination IO plus non-IO therapy group (*n* = 10), the regimens were the following: anti-PD-L1/poly-ADP ribose polymerase (PARP) inhibitors (*n* = 3), anti-PD-L1/anti-angiogenic tyrosine kinase inhibitors (*n* = 2), anti-PD-1/PARP/VEGF inhibitors (*n* =1), anti-CTLA-4 inhibitor/anti-angiogenic therapy (*n* = 1), anti-PD-1/PI3K-gamma inhibitors (*n* = 1), and chemotherapy either with anti-PD-1/IDO-1 inhibitors (*n* = 1) or with pegylated IL-10 (*n* = 1). There were no differences in baseline clinicopathologic characteristics between the three groups (Table 1).

### 3.1. Toxicity

Between the three groups, there were no differences in TRAEs of any grade (*p* = 0.437), grade 3 or 4 TRAEs (*p* = 0.082), or immune-related adverse events (irAEs) (*p* = 0.110). In the monotherapy group, any-grade TRAEs occurred in 5 of 8 patients (62.5%), grade 3/4 TRAEs in 1 patient (12.5%), and irAEs in 1 patient (12.5%). In the combination IO-only group, any-grade TRAEs, grade 3/4 TRAEs, and irAEs occurred in 13 (76.5%), 1 (5.9%), and 5 (29.4%) of 17 patients, respectively. In the combination IO plus non-IO group, any-grade TRAEs, grade 3/4 TRAEs, and irAEs occurred in 9 (90.0%), 3 (30.0%), and 6 (60.0%) of 10 patients, respectively.

### 3.2. Clinical Efficacy and Survival

**Figure 1:**
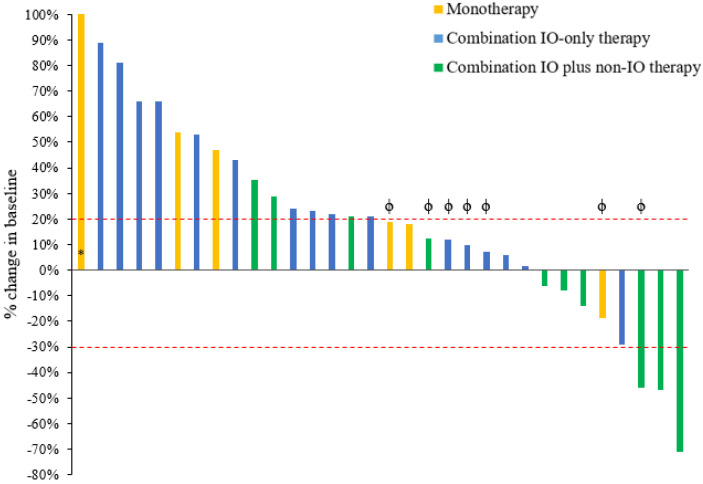
Radiologic response to immunotherapy in patients with MSS endometrial cancer. This waterfall plot illustrates the best objective response in 32 evaluable patients treated with immunotherapy using RECIST 1.1 criteria. Each bar represents a patient and shows the maximum percentage change from baseline in the sum of the longest diameters of all target lesions while on immunotherapy. The area above the upper red dotted line represents progressive disease (≥20% increase in the sum of the diameters of the target lesions compared with the baseline). The area between both upper and lower red dotted lines represents stable disease. The area below the lower red dotted line represents treatment response (≥30% decrease in the sum of the diameters of the target lesions compared with the baseline). ^ϕ^ Despite the change in the sum of the target lesions diameter not meeting the criteria of progressive disease, patients indicated by “ϕ” were classified as progressive disease by RECIST 1.1 criteria if there was growth of non-target lesions or the appearance of new lesions; this occurred in 2 patients with monotherapy, 3 patients with combination IO only, and 2 patients with combination IO plus non-IO therapy. * This patient treated with monotherapy had a 292% increase in the sum of the diameters of the target lesions compared with baseline. IO = immuno-oncology.

**Figure 2:**
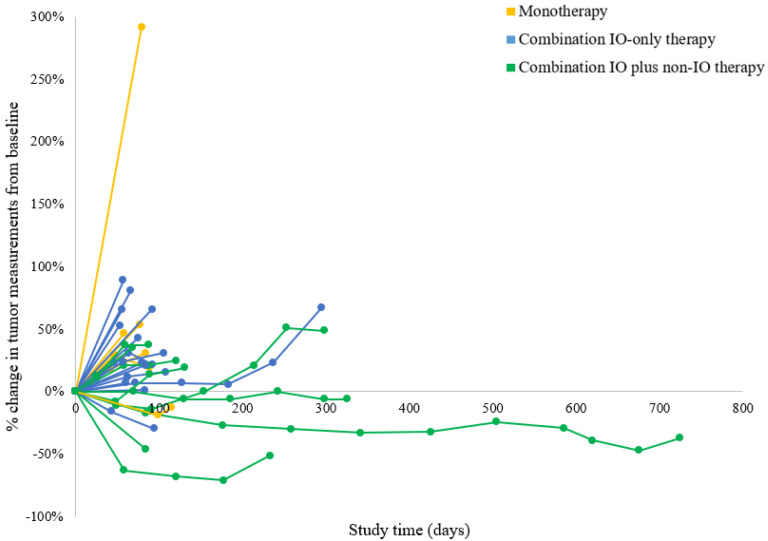
Tumor response across time. This spider plot demonstrates the tumor measurements from baseline using RECIST 1.1 criteria during the course of treatment with immunotherapy in 32 evaluable patients. IO = immuno-oncology.

Table 2 demonstrates the objective radiologic response to treatment in the 32 evaluable patients. Three patients (two receiving monotherapy and one receiving combination IO-only therapy) were removed from the study (worsening performance status or withdrawal of consent) prior to the first radiologic response assessment; therefore, their responses were non-evaluable. Figure 1 shows each patient’s greatest percentage change in target tumor lesions, and Figure 2 shows the dynamic tumor volume changes across time. The monotherapy, combination IO-only, and combination IO plus non-IO groups had no statistically significant difference in ORR (0% vs. 0% vs. 10%, respectively; *p* = 0.500). The responding patient was on a regimen of anti-PD-L1/PARP/VEGF inhibitors and had a partial response that lasted 15.3 months. This patient had a grade 2 MSS tumor of endometrioid histology and no *BRCA* mutations. Somatic gene panel testing for this patient’s tumor demonstrated *PTEN* (c.306del) and two activating *PIK3CA* (c.328_330del and c.1616C > G) mutations.

No statistically significant differences in CBR were seen between the three groups (16.7% vs. 18.8% vs. 50%, respectively; *p* = 0.197). However, the 3 patients who had the longest duration of clinical benefit were in the combination IO plus non-IO group; 1 was treated with anti-PD-L1/PARP/VEGF inhibitors (duration of benefit, 21.0 months), 1 with anti-PD-L1/PARP inhibitors (8.4 months), and 1 with anti-PD-L1/anti-angiogenic tyrosine kinase inhibitors (8.1 months) (Table 3). The remaining 2 patients who had clinical benefit in the combination IO plus non-IO group were individually treated with anti-PD-L1/PARP inhibitors (5.8 months) and anti-PD-L1/anti-angiogenic tyrosine kinase inhibitors (2.7 months). The duration of disease control for patients treated with monotherapy or combination IO-only therapy was transient, except for 1 patient treated with anti–PD-L1/OX40 monoclonal antibodies (7.4 months).

**Figure 3:**
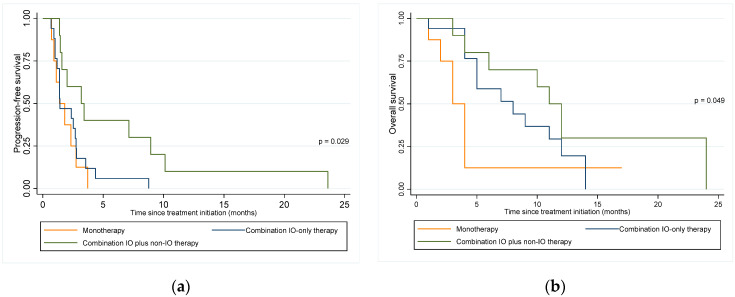
Kaplan–Meier survival curves. (**a**) Progression-free survival based on drug regimen; (**b**) Overall survival based on drug regimen. IO = immune-oncology.

The overall median follow-up among the patients who were alive at the time of evaluation was 12.5 months (range 5–18). For patients who were deceased at the time of evaluation, the median follow-up was 5 months (range 1–24). All 35 patients had disease progression, and 29 patients (82.9%) died. Figure 3 demonstrates the Kaplan–Meier curves for PFS and OS based on type of treatment regimen. The median PFS durations for the monotherapy, combination IO-only therapy, and IO plus non-IO therapy groups were 1.4 (95% CI 0.72–2.76), 1.4 (95% CI 1.0–2.8), and 3.2 (95% CI 1.4–8.9) months, respectively. Patients who were treated with combination IO plus non-IO therapy were observed to have significantly improved PFS compared to those treated with monotherapy (HR 0.56, 95% CI 0.33–0.97; *p* = 0.037) or combination IO-only therapy (HR 0.36, 95% CI 0.15–0.90; *p* = 0.028). Adjusting for prior lines of therapy, PFS was still significantly improved in the combination IO plus non-IO group compared to the combination IO-only group (HR 0.32, 95% CI 0.12–0.88; *p* =0.027). There was a trend towards improved PFS for the combination IO plus non-IO group compared to the monotherapy group (HR 0.60, 95% CI 0.34–1.1; *p* = 0.083). There were no statistically significant differences in PFS when comparing the monotherapy and combination IO-only therapy groups with or without adjustment for prior lines of therapy (*p* = 0.586 and 0.501, respectively). The median OS durations for the monotherapy, combination IO-only therapy, and IO plus non-IO therapy groups were 3 (95% CI 1–4), 8 (95% CI 4–12), and 11 (95% CI 3–not reached) months, respectively. There was a trend in improved OS for patients treated with combination IO plus non-IO therapy compared to those treated with monotherapy (HR 0.60, 95% CI 0.35–1.0; *p* = 0.068). When adjusting for prior lines of therapy, there was a statistically significant improvement in OS for the combination IO plus non-IO therapy group compared to the monotherapy group (HR 0.50, 95% CI 0.27–0.95; *p* = 0.036). There was no statistically significant difference in OS between the combination IO-only therapy group and the combination IO plus non-IO therapy group with (HR 0.54, 95% CI 0.21–1.4; *p* = 0.213) or without adjustment (HR 0.53, 95% CI 0.21–1.33; *p* = 0.178) for prior lines of therapy. There were no statistically significant differences in OS when comparing the monotherapy and combination IO-only therapy groups with or without adjustment for prior lines of therapy (*p* = 0.103 and 0.154, respectively).

## 4. Discussion

The advent of immunotherapy has dramatically changed the landscape of oncology treatment and presents opportunities to improve the treatment of recurrent endometrial cancer. We reviewed the characteristics and clinical outcomes of patients with recurrent MSS endometrial cancer undergoing immunotherapy treatment in early-phase clinical trials during a 10-year period to identify better rational therapeutic regimens and combinations in this population. Despite the treatment heterogeneity and overall poor objective radiologic responses, there are several notable observations.

As anticipated, single-agent immune checkpoint blockade was observed to have limited efficacy in recurrent MSS endometrial tumors in this study. All patients who received monotherapy either had disease progression as their best objective response (*n* = 7) or progressed quickly after (*n* = 1; stable disease for 1.4 months). Patients who received combination IO-only therapy were mainly treated with doublet or triplet immune checkpoint therapy and had similar results. Regardless of affecting multiple independent, complementary immune checkpoint pathways, there were no responders, and only 1 patient (treated with anti-PD-L1 inhibitor/OX40 agonist) achieved durable disease control of 7.4 months. Interestingly, increasing the number of immune checkpoint inhibitors did not necessarily improve efficacy as none of the patients who received triplet immune checkpoint therapy had disease control. Additionally, compared to patients who were treated with monotherapy, patients receiving combination IO-only therapy did not have improved PFS or OS.

For patients treated with combination IO plus non-IO therapy, although there was only 1 partial responder in the cohort, the clinical benefit rate was 50%, with 4 patients demonstrating among the longest durations of disease control in this series (21.0, 8.4, 8.1, and 5.8 months). This cohort also demonstrated the best anti-tumor activity as shown by the 3 greatest reductions in tumor target lesions being in this cohort. Additionally, patients treated with combination IO plus non-IO therapy had favorable survival outcomes. PFS was significantly improved in patients treated with combination IO plus non-IO therapy compared to those treated with monotherapy or combination IO-only therapy. Given a trend toward difference in number of prior lines of systemic therapy between groups, we adjusted for this variable in the Cox proportional hazards model, and trends in PFS benefit were still observed. Furthermore, the combination IO plus non-IO therapy group had significantly improved OS compared to the monotherapy group when adjusting for prior lines of treatment.

There are several possible explanations for the limited efficacy of monotherapy and combination IO-only therapy (specifically immune checkpoint therapy) in this series. MSS tumors are typically known to have low tumor mutational burden compared to their MSI-H counterparts; this results in fewer produced neoantigens, lower tumor immunogenicity, and less infiltration by tumor-infiltrating lymphocytes, which ultimately result in poorer response to immune checkpoint therapy [14,15,16,17]. Furthermore, many endometrial tumors have inactivation of the tumor suppressor PTEN, resulting in increased PI3K signaling and subsequently higher VEGF production, reduced TIL infiltration, and an immunosuppressed tumor microenvironment [11,18]. Additionally, immunosuppressive tumor microenvironments may lead to conditions where these sub-primed T cells become dysfunctional or apoptotic when stimulated by immune checkpoint therapy [19,20].

In contrast, agents that help modify the tumor microenvironment may improve IO therapy, and this may help explain the greater tumor reductions and disease control in the combination IO plus non-IO cohort [18,21]. Patients who had the greatest duration of clinical benefit in this group were treated with an anti-PD-L1 inhibitor in combination with PARP, VEGF, or anti-angiogenic tyrosine kinase inhibitors. Although PARP inhibitors have been utilized in HR-deficient tumors (e.g., ovarian tumors with *BRCA* mutations), there is evidence that PTEN plays a role in the HR pathway through upregulation of RAD51, thereby decreasing double-stranded breaks and maintaining genomic stability [22,23,24]. Given the frequent loss of PTEN in endometrial cancers, PARP inhibitors may have utility in these tumors [23,24]. Furthermore, PARP inhibitors may increase tumor immunogenicity via immunogenic cell death and expansion of the neoantigen repertoire, cGAS-STING pathway activation, upregulation of PD-L1 expression, and immunomodulation of the tumor microenvironment to reflect a T_H_1 immune response [25,26,27,28,29]. This may explain the durable clinical benefit in 3 patients. It should be noted that the somatic *BRCA1* mutations in 2 patients treated with PARP inhibitors may also have contributed to improved response to the PARP inhibitor combination. Anti-angiogenic therapy (e.g., VEGF or tyrosine kinase inhibitors) may improve the efficacy of IO therapy given that tumor-associated angiogenesis is linked with immunosuppression in the tumor microenvironment [30]. VEGF can result in immunosuppression via inhibition of cytotoxic T cell trafficking/fitness and dendritic cell maturation [31,32,33]. Furthermore, VEGF promotes infiltration of immunosuppressive myeloid-derived suppressor cells and type II tumor-associated macrophages [33,34]. Recently, the combination of anti-PD-1 inhibitors and tyrosine kinase inhibitors with anti-angiogenic activity has demonstrated efficacy in MSS recurrent endometrial cancer. In a phase II trial of 102 advanced/recurrent endometrial cancers (KEYNOTE-146), Makker et al. demonstrated that the combination of pembrolizumab and lenvatinib (a tyrosine kinase inhibitor with multiple targeted effects, including blocking VEGFR1-3) had an ORR of 38% [35]. This result was impressive given that the study population consisted of predominantly MSS tumors and that high-grade serous tumors responded as well (ORR 40%) [35].

The current study has several strengths. First, this study compares the objective response and survival data between various immunotherapy regimens over a 10-year time period in one of the largest clinical trial centers. Furthermore, the study population had a higher proportion of non-White study participants compared to what has been reported in the literature for early-phase gynecologic oncology trials (42.9% vs. 21%) [36]. Historically, clinical trial populations have consisted of predominantly White study participants, and more representative demographics are critical to unravelling the racial differences in tumor biology and response to treatment [36,37]. There are several study limitations. Given heterogeneous regimens within the 3 treatment groups, it is difficult to perform subgroup analyses of the treatment regimens using statistical methods due to small numbers. The IO therapies presented in this series consisted of mainly immune checkpoint therapies (particularly anti-PD-1, anti-PD-L1, and anti-CTLA inhibitors) and therefore these findings are not generalizable to other IO therapies such as cell therapies, vaccines, or cytokines. Additionally, further translational data (e.g., tumor mutational burden or TIL counts) would help to clarify whether certain intrinsic tumor or tumor microenvironment factors played a role in the observed varying degrees of anti-tumor activity in this series.

## 5. Conclusions

In summary, patients with recurrent MSS endometrial cancer treated with monotherapy or combination immune checkpoint-only therapy demonstrated worse survival outcomes than those treated with combination IO plus non-IO therapy. Although there was only one objective response, durable clinical benefit was seen in some patients when combining immune checkpoint therapy with PARP inhibitors or anti-angiogenic therapy. These results highlight the importance of selecting rational combinational immunotherapy regimens for clinical trials in MSS endometrial cancer. Future studies will be needed to identify patients who will benefit the most from adjunctive PARP inhibitor and anti-angiogenic therapeutics.

## Figures and Tables

**Table 1:** Clinical characteristics and treatment regimens for patients with MSS endometrial cancer (*n* = 35).

	Overall (*n* = 35)	Monotherapy (*n* = 8)	Combination		*p*-Value
			IO-Only Therapy (*n* = 17)	IO Plus Non-IO Therapy (*n* = 10)	
Age (median, range), years	64 (37–73)	65 (54–73)	64 (37–71)	56 (44–68)	0.2111 ^k^
Race/ethnicity					0.309 ^f^
Non-Hispanic White	20 (57.1%)	7 (87.5%)	8 (47.1%)	5 (50.0%)	
Black	6 (17.1%)	0 (0.0%)	5 (29.4%)	1 (10.0%)	
Hispanic	5 (14.3%)	0 (0.0%)	2 (11.8%)	3 (30.0%)	
Asian	4 (11.4%)	1 (12.5%)	2 (11.8%)	1 (10.0%)	
ECOG performance status					>0.999 ^f^
0	1 (2.9%)	0 (0.0%)	1 (5.9%)	0 (0.0%)	
1	34 (97.1%)	8 (100%)	16 (94.1%)	10 (100%)	
MSI status					0.229 ^f^
MSS	34 (97.1%)	7 (87.5%)	17 (100%)	10 (100%)	
MSI unknown	1 (2.9%)	1 (12.5%)	0 (0.0%)	0 (0.0%)	
Histologic type					0.357 ^f^
Endometrioid	15 (42.9%)	2 (25.0%)	6 (35.3%)	7 (70.0%)	
Serous	8 (22.9%)	3 (37.5%)	4 (23.5%)	1 (10.0%)	
Clear cell	5 (14.3%)	1 (12.5%)	4 (23.5%)	0 (0.0%)	
Carcinosarcoma	4 (11.4%)	1 (12.5%)	1 (5.9%)	2 (20.0%)	
Mixed	3 (8.6%)	1 (12.5%)	2 (11.8%)	0 (0.0%)	
Tumor grade					0.832 ^f^
2	5 (14.3%)	1 (12.5%)	2 (11.8%)	2 (20.0%)	
3	30 (85.7%)	7 (87.5%)	15 (88.2%)	8 (80.0%)	
Prior treatment					0.1428 ^k^
Lines of systemic therapy (median, range)	3 (1–10)	3 (2–7)	4 (1–10)	2 (1–5)	
Number of immune checkpoint inhibitors					<0.001 ^f^
None	1 (2.9%)	0 (0.0%)	0 (0.0%)	1 (10.0%)	
1	19 (54.3%)	8 (100%)	2 (11.8%)	9 (90.0%)	
2	12 (34.3%)	0 (0.0%)	12 (70.6%)	0 (0.0%)	
3	3 (8.6%)	0 (0.0%)	3 (17.6%)	0 (0.0%)	

Abbreviations: ECOG, Eastern Cooperative Oncology Group; IO, immuno-oncology; MSI, microsatellite instability; MSS, microsatellite stable. ^f^ Fisher’s exact test, ^k^ Kruskal–Wallis test.

**Table 2:** Evaluable responses (*n* = 32) to immunotherapy treatment in MSS endometrial cancer.

Response Category	Monotherapy (*n* = 6)	Combination Therapy		*p*-Value
		IO Only (*n* = 16)	IO Plus Non-IO (*n* = 10)	
PR	0 (0%)	0 (0%)	1 (10.0%)	0.337 ^f^
CR	0 (0%)	0 (0%)	0 (0.0%)	
SD	1 (16.7%)	3 (18.8%)	4 (40.0%)	
PD	5 (83.3%)	13 (81.2%)	5 (50.0%)	
ORR	0 (0%)	0 (0%)	1 (10.0%)	0.500 ^f^
CBR	1 (16.7%)	3 (18.8%)	5 (50.0%)	0.197 ^f^

Abbreviations: CBR, clinical benefit rate; CR, complete response; IO, immuno-oncology; MSS, microsatellite stable; ORR, objective response rate; PD, progressive disease; PR, partial response; SD, stable disease. Objective response was defined as PR or CR. Clinical benefit was defined as PR, CR, or SD. ^f^ Fisher’s exact test.

**Table 3:** Patients who had disease control on immunotherapy.

Regimen	Group	Histo	Duration of Clinical Benefit (Months)	OS (Months)	Mutations
-Anti-PD-L1 inhibitor -PARP inhibitor -VEGF inhibitor	IO plus non-IO	E	21.0	24	Inactivating: *PTEN* (c.306del) Activating: *PIK3CA* (c.328_330del and c.1616C > G)
-Anti-PD-L1 inhibitor -PARP inhibitor	IO plus non-IO	S	8.4	12	Inactivating: *BRCA1* (c.1513) and *TP53* (p.R273H) Unknown functional significance: *RAD54L*, *ESR1*, *SYK*, *MAP3K1*, and *MLL*
-Anti-PD-L1 inhibitor -Tyrosine kinase inhibitor	IO plus non-IO	E	8.1	18	Inactivating: *PTEN* (c.697C > T) and *FBXW7* (c.2065C > T) Activating: *KRAS* (c.35G > C)
-Anti-PD-L1 inhibitor -OX40 agonist	Combo IO	M	7.4	14	Inactivating: *FBXW7* (c.1514G > A) Benign: *TP53*
-Anti-PD-L1 inhibitor -PARP inhibitor	IO plus non-IO	CS	5.8	12	Inactivating: *PTEN* (c.955_958del), *BRCA1* (c.3013G > T), and *PIK3R1* (c.1393_1401del) Unknown functional significance: *AXL* and *NF1*
-Anti-PD-L1 inhibitor -Tyrosine kinase inhibitor	IO plus non-IO	E	2.7	3.4	Inactivating: *PTEN* (c.388C > T and c.295G > T p.E99), *TP53* (c.578A > G), and *PIK3R1* (c.1699A > G) Unknown functional significance: *CTNNB1*
-Anti-CTLA-4 inhibitor -TLR agonist	Combo IO	S	1.7	5	Inactivating: *PIK3R1* (c.1727_1729del), *TP53* (c.581T > G), and *PI3KR1* (c.1112del) Unknown functional significance: *FBXW7*
-Anti-PD-1 inhibitor	Mono	E	1.4	4	Unknown functional significance: *TP53*
-Anti-PD-1 inhibitor -Anti-PD-L1 inhibitor	Combo IO	E	0.9	5	Inactivating: *PTEN* (c.388C > G) and *TP53* (c.659A > G) Unknown functional significance: *PIK3R1*, *RNF43*, and *FBXW7*

Abbreviations: Combo IO, Combination IO only therapy; CS, carcinosarcoma; E, endometrioid; Histo, histology; IO, immuno-oncology; IO plus non-IO, combination IO plus non-IO therapy; M, mixed histology; Mono, monotherapy; OS, overall survival; S, serous.

## Data Availability

The datasets used and/or analyzed during the current study are available from the corresponding author on reasonable request and according to available guidelines at time of request.
